# Apple Yield Estimation Method Based on CBAM-ECA-Deeplabv3+ Image Segmentation and Multi-Source Feature Fusion

**DOI:** 10.3390/s25103140

**Published:** 2025-05-15

**Authors:** Wenhao Cui, Yubin Lan, Jingqian Li, Lei Yang, Qi Zhou, Guotao Han, Xiao Xiao, Jing Zhao, Yongliang Qiao

**Affiliations:** 1 College of Agricultural Engineering and Food Science, Shandong University of Technology, Zibo 255000, China; 15053648985@163.com (W.C.); ylan@sdut.edu.cn (Y.L.); 19811715026@163.com (J.L.); 23403010305@stumail.sdut.edu.cn (L.Y.); 18095219962@163.com (Q.Z.); 17852030301@163.com (G.H.); 23403010293@stumail.sdut.edu.cn (X.X.); 2 Shandong Provincial Engineering Technology Research Center for Agricultural Aviation Intelligent Equipment, Zibo 255049, China; 3 Australian Institute of Machine Learning (AIML), The University of Adelaide, Adelaide, SA 5000, Australia

**Keywords:** apple yield estimation, semantic segmentation, CBAM-ECA-DeepLabv3+ model, UAV remote sensing imagery, multi-source feature fusion, support vector machine

## Abstract

Apple yield estimation is a critical task in precision agriculture, challenged by complex tree canopy structures, growth stage variability, and orchard heterogeneity. In this study, we apply multi-source feature fusion by combining vegetation indices from UAV remote sensing imagery, structural feature ratios from ground-based fruit tree images, and leaf chlorophyll content (SPAD) to improve apple yield estimation accuracy. The DeepLabv3+ network, optimized with Convolutional Block Attention Module (CBAM) and Efficient Channel Attention (ECA), improved fruit tree image segmentation accuracy. Four structural feature ratios were extracted, visible-light and multispectral vegetation indices were calculated, and feature selection was performed using Pearson’s correlation coefficient analysis. Yield estimation models were constructed using k-nearest neighbors (KNN), partial least squares (PLS), random forest (RF), and support vector machine (SVM) algorithms under both single feature sets and combined feature sets (including vegetation indices, structural feature ratios, SPAD, vegetation indices + SPAD, vegetation indices + structural feature ratios, structural feature ratios + SPAD, and the combination of all three). The optimized CBAM-ECA-DeepLabv3+ model achieved a mean Intersection over Union (mIoU) of 0.89, an 8% improvement over the baseline DeepLabv3+, and outperformed U2Net and PSPNet. The SVM model based on multi-source feature fusion achieved the highest apple yield estimation accuracy in small-scale orchard sample plots (R^2^ = 0.942, RMSE = 12.980 kg). This study establishes a reliable framework for precise fruit tree image segmentation and early yield estimation, advancing precision agriculture applications.

## 1. Introduction

Early acquisition of orchard yield information can assist growers in the timely adjustment of production strategies, optimization of resource allocation, rational harvest planning, and orderly deployment of labor and capital [1]. Traditional fruit yield estimation methods mainly rely on manual counting and weighing, which are labor-intensive, inefficient, and difficult to apply in large-scale orchards, thus failing to meet the demands of precision management in modern agriculture. Although yield prediction models have been extensively explored in field crops such as rice, maize, and wheat [2], research on woody crops remains relatively limited, especially at the block-scale yield estimation level [3].

Machine vision systems based on image processing and deep learning have developed rapidly in the agricultural field and have been widely applied in orchard yield prediction [4]. Early fruit detection and yield estimation methods mostly relied on traditional image processing techniques or machine learning classification models based on color, shape, and texture features. For example, Dorj et al. [5] identified and counted citrus fruits based on color features, achieving a correlation coefficient of 0.93 with manually annotated results. Sun et al. [6] fused RGB color features and depth information to achieve real-time localization and 3D modeling of green apples in complex orchard environments, providing accurate data support for fruit yield estimation. However, image processing methods based on color features face limitations in dealing with complex scenes such as fruit overlapping, branch occlusion, and illumination variations, often resulting in missed detections or false positives [7]. Deep learning-based image processing algorithms can mitigate these issues. For instance, Jia et al. [8] embedded an attention mechanism into Mask R-CNN to suppress noise caused by occlusions and overlaps, Bai et al. [9] introduced a CBAM into the U-Net architecture to enhance winter flush extraction for litchi, and Li et al. [10] proposed a multi-view channel attention and random interpolation enhancement method, significantly improving the recognition and segmentation performance of minor features under small sample conditions. Koirala et al. [11] provided a comprehensive review of deep learning technologies in fruit detection and yield estimation. Apolo-Apolo et al. [12] combined Faster R-CNN and LSTM models to achieve time-series-based citrus yield estimation, with an estimation error controlled within 7.22%. Li et al. [13] improved the lightweight YOLOv5 network and integrated a yield fitting model, achieving rapid and stable apple yield estimation at the single-tree level, with a relative error range within 7%. Kestur et al. [14] developed MangoNet, a deep convolutional neural network based on semantic segmentation combined with contour detection, demonstrating robust fruit detection performance under complex environmental conditions and providing a data foundation for subsequent mango yield estimation. These methods mostly focus on the fruit maturity stage, rely on a single data type, or are limited to the scale of individual trees, which constrains their applicability for large-scale yield estimation.

Remote sensing technologies have demonstrated strong potential for monitoring agricultural variables, including vegetation health, biomass, and yield prediction [15]. Vegetation indices derived from multispectral or hyperspectral imagery, such as NDVI and EVI, are among the most widely used features in remote sensing applications. These indices provide valuable information for assessing crop status and have been widely adopted in precision agriculture practices. However, studies specifically focusing on fruit yield estimation using remote sensing data remain limited. Suarez et al. [16] utilized time-series remote sensing data combined with machine learning models to predict block-scale citrus yield in Australian orchards; Gómez-Lagos et al. [17] integrated multi-temporal NDVI, spatial fuzzy C-means clustering, and neural networks to achieve small block-scale yield estimation in vineyards. When using spectral information alone for yield estimation, the accuracy may be affected by classification errors and information loss during feature extraction [18,19]. However, integrating spectral features with information such as structural morphology, climate parameters, and topographic factors can effectively improve the robustness of the model [20]. Liakos et al. [21] further reviewed the application of various machine learning methods in agriculture, emphasizing that multimodal data fusion can significantly improve prediction accuracy and practicality. Cao et al. [22] integrated meteorological, soil, and remote sensing spectral data and applied machine learning and deep learning algorithms to achieve high-precision yield prediction at a regional scale; Chen et al. [23] fused LiDAR and multispectral data to build a yield estimation model based on canopy volume, vegetation indices, and projection area, achieving an R^2^ of 0.758; and Sun et al. [24] combined point cloud features and vegetation indices using a neural network to predict apple yield, with R^2^ values ranging from 0.83 to 0.88.

This study proposes a multi-source feature fusion model for apple yield estimation, integrating spectral features (vegetation indices derived from UAV remote sensing imagery), structural features (proportional ratios of flowers, fruits, leaves, and branches extracted from ground-based fruit tree images), and biochemical features (leaf chlorophyll content measured as SPAD values) to enhance the accuracy and robustness of yield prediction in small-scale orchard sample plots. The main contributions of this study are as follows: (1) A systematic evaluation of three mainstream semantic segmentation models (U2Net, PSPNet, and DeepLabv3+) for recognizing tree structural organs (flowers, fruits, leaves, and branches) in orchard environment images, with the optimal DeepLabv3+ model enhanced by integrating Convolutional Block Attention Module (CBAM) and Efficient Channel Attention (ECA) mechanisms to achieve superior segmentation accuracy; (2) Extraction of structural feature ratios (proportions of flowers, fruits, leaves, and branches) from segmentation results, which are fused with spectral features (vegetation indices from UAV remote sensing imagery) and biochemical features (SPAD values) to form a multi-source feature set, comprehensively characterizing tree growth status and yield potential across spectral, structural, and biochemical dimensions; (3) Yield estimation models based on KNN, PLS, RF, and SVM algorithms are constructed using single-feature, dual-source fusion, and multisource fusion approaches, and the effects of different feature combinations on yield estimation accuracy are systematically analyzed to determine the optimal modeling strategy suitable for small block-scale orchard yield prediction.

## 2. Materials and Methods

### 2.1. Experimental Site

**Figure 1:**
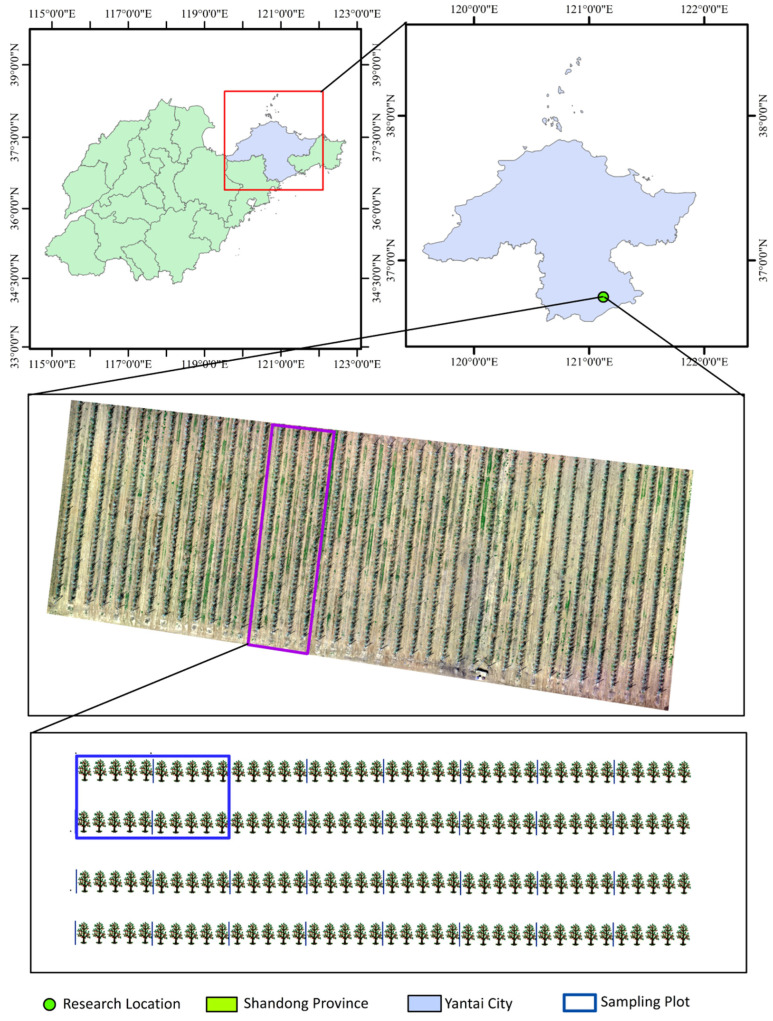
Map of the experimental area.

The experimental area is located in a modern apple orchard (36°48′55″ N, 121°14′40″ E) in Haiyang City, Shandong Province, China. The site has an average altitude of approximately 200 m, an average annual temperature of about 12 °C, an average annual rainfall of 695.7 mm, and an average annual relative humidity of 68%. The region is characterized by a temperate, semi-humid continental monsoon climate. The total area of the selected plot is 23 m × 136 m, with 40 rows of apple trees planted. The plot is divided into 80 sampling areas, each approximately 6 m × 4.5 m in size, containing two rows of apple trees with 10 trees in each row, as shown in Figure 1.

### 2.2. Data Collection and Processing

#### 2.2.1. Multi-Source Ground Data Acquisition of Apple Tree

**Figure 2:**
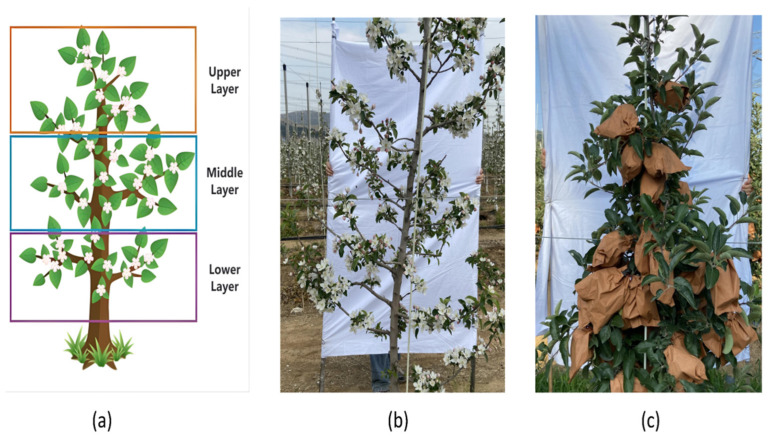
Multi-source data acquisition method of apple tree. (**a**) Leaf SPAD sampling. (**b**) Flowering stage images. (**c**) Young fruit stage images.

Multi-source ground data included apple yield, tree images, and chlorophyll relative content (SPAD values). As a critical biochemical indicator influencing photosynthetic capacity and regulating tree productivity, SPAD values were measured using a MultispeQV2 multifunctional plant meter (PhotosynQ Inc., East Lansing, MI, USA) during both the flowering and young fruit stages. In each sampling plot, three trees were randomly selected. Two mature leaves were sampled from the upper, middle, and lower canopy layers from each tree, respectively. The average of six measurements per tree was recorded as the final SPAD value (Figure 2a).

During the flowering and young fruit stages of apple trees, smartphone images of the south-facing side (receiving more prolonged sunlight exposure than the northern side) were captured under natural ambient lighting without direct sunlight. The smartphone was positioned parallel to the tree trunk, at a distance of 1 m from the tree and 1.2 m above ground level, with a white backdrop (1.8 m × 1 m) placed behind the tree. Three fruit trees were randomly photographed in each sampling plot. A total of 480 apple tree images were collected in the two periods, as shown in Figure 2b,c.

During the data acquisition process, samples were collected at two critical growth stages of apple trees: the flowering stage and the young fruit stage. These two phenological phases exhibit significant disparities in canopy architecture, leaf density, and fruit visibility, resulting in variations in feature distributions, such as SPAD values, vegetation indices, and fruit tree structural feature ratios. These stage-specific differences directly impact model training and can introduce heterogeneity into the dataset. Consequently, by incorporating samples from both growth stages into the dataset and by designing features that implicitly capture developmental variations, the model was enabled to generalize across different growth phases, thereby enhancing its robustness and predictive accuracy [25].

Apple yield data were collected on 30 October 2023. In each sampling plot, five fruit trees were randomly selected, and all fruits from these trees were harvested for counting and weighing. The average number of fruits per tree and the average weight of a single apple were calculated based on these five trees. The yield per tree was estimated by multiplying the two averages, and the total yield of each plot was then calculated from this value.

#### 2.2.2. UAV Remote Sensing Image Acquisition and Preprocessing

This study utilized two unmanned aerial vehicles (UAVs) to acquire orchard imagery during the young fruit stage of apple development: a DJI M300 (DJI, Shenzhen, China) equipped with a P1 camera (DJI, Shenzhen, China) (35 mm visible-light lens) for visible-light imaging, and a DJI M210 (DJI, Shenzhen, China) carrying a Changguang Yuchen MS600 Pro multispectral camera (Yusense, Inc., Qingdao, China) for multispectral data acquisition. The configurations of the UAV and camera parameters are detailed in Table 1. Remote sensing data collection was conducted between 10:00 and 14:00 local time under clear and windless conditions. Pix4Dmapper software (version 4.5.6) was utilized for the stitching and processing of both visible-light and multispectral imagery.

#### 2.2.3. Fruit Tree Image Preprocessing

**Figure 3:**
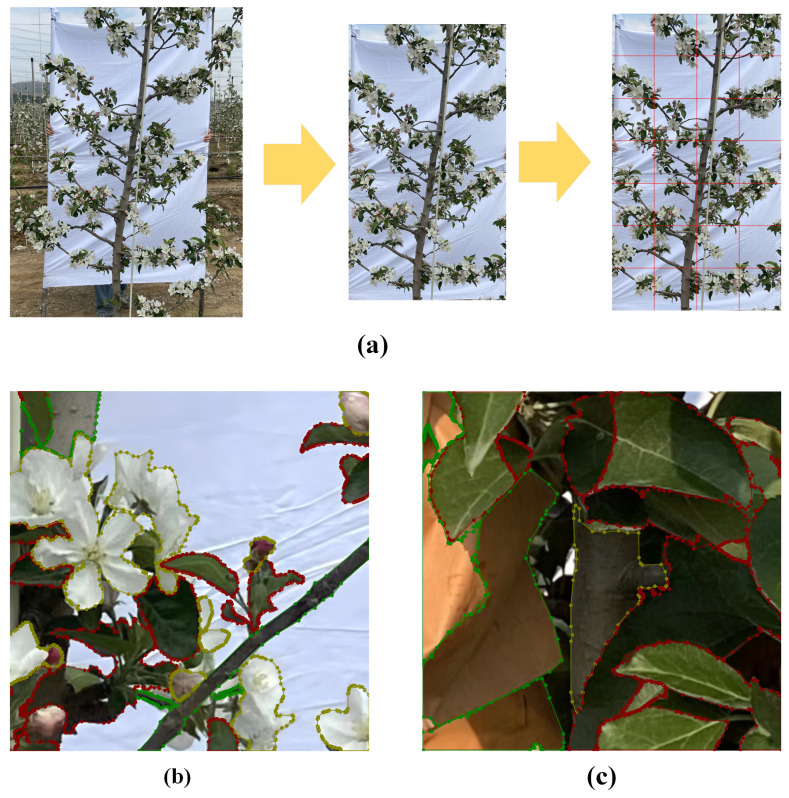
Preprocessing of apple tree images. (**a**) Example of image cropping and segmentation. (**b**) Examples of flowering period labeling. (**c**) Examples of young fruit stage labeling.

The edges of the apple tree images were cropped using Lightroom software (version 9.0), retaining only the portions containing fruit trees against a white background (Figure 3a). Prior to annotation, the cropped images were programmatically divided into smaller sections of 512 × 512 pixels to enhance the precision of classification. After manually screening and removing images that contained only white background, a total of 2000 images were obtained for each stage—the flowering and young fruit stages. Image annotation was performed using the Labelme tool. For images of the flowering stage, the annotation objects were green leaves, flowers, and tree trunks (Figure 3b). For images at the young fruit stage, the annotated objects were green leaves, bagging (used to cover young fruits and thus treated as a fruit annotation), and tree trunks (Figure 3c). During annotation, the outline of each object was followed, and irregular bounding boxes were drawn according to the actual position of each fruit tree organ, with corresponding labels assigned.

### 2.3. Selection of Semantic Segmentation Model

Image semantic segmentation aims to partition an image into distinct regions, enabling the recognition and understanding of various objects within it. In this paper, U2-Net [26], PSPNet [27], and DeepLabv3+ [28] were employed to perform the segmentation of the flowers, fruits, leaves, and branches of apple trees. The model that achieved the highest segmentation accuracy was selected and further refined to enhance overall performance.

U2-Net adopts a U-shaped dual encoder–decoder architecture, embedding a smaller U-Net structure within the main U-Net to strengthen global and local feature extraction capabilities. It effectively captures features at multiple scales while maintaining computational efficiency, demonstrating superior performance with relatively low computational cost. The Pyramid Scene Parsing Network (PSPNet) is a deep learning model designed for semantic segmentation, which aggregates contextual information from different scales through a unique pyramid pooling module to capture global features. This approach enables precise pixel-level segmentation, making PSPNet particularly effective for interpreting complex scenes and improving segmentation performance. DeepLabv3+ employs an encoder–decoder architecture that incorporates atrous convolution and an Atrous Spatial Pyramid Pooling (ASPP) module to extract multi-scale features and refine edge details. It enhances segmentation accuracy while preserving spatial resolution, making it well-suited to processing complex backgrounds and small objects.

### 2.4. Image Semantic Segmentation Model Training

The model training environment for this study was configured as follows: an Intel Core i9-9820X (Intel Corporation, Santa Clara, CA, USA) processor (3.3 GHz), 32 GB of random-access memory (RAM), and an NVIDIA (NVIDIA Corporation, Santa Clara, CA, USA) GeForce RTX 2080 Ti graphics processing unit (GPU) with 11 GB of memory, running on Ubuntu 18.04 LTS. The training framework was based on PyTorch (version 1.13), with CUDA used to accelerate the training process. The dataset comprised 2000 images of apple trees at the flowering stage and 2000 images at the young fruit stage, resulting in a total of 4000 images. To ensure class balance, the dataset was divided into training and test sets with a 4:1 ratio for both flowering and young fruit stage images. All models were trained under the same hyperparameter settings: epochs = 300; batch size = 8; learning rate = 0.01.

### 2.5. Extraction and Selection of Characteristic Parameters of Apple Yield Estimation Model

To accurately estimate apple yield, three types of features were extracted: visible-light and multispectral vegetation indices, structural feature ratios of fruit trees, and relative chlorophyll content. Features that exhibited a high correlation with apple yield were selected to provide input data for the subsequent development of the yield estimation model.

#### 2.5.1. Calculation of Visible and Multispectral Vegetation Index

Vegetation indices (VIs) are a series of indices derived from the mathematical calculation of reflectance values in specific spectral bands, based on the varying spectral responses of green vegetation. The visible light vegetation index (VARI), as selected in this study, can reduce atmospheric interference and enhance the stability of the vegetation index. ExR and ExG are sensitive to chlorophyll content. GLI primarily reflects chlorophyll content and is sensitive to the physiological state of vegetation. NDI can be applied to different vegetation types and growth stages. MGRVI has higher sensitivity and anti-interference ability. The multispectral vegetation index (DVI) reflects vegetation growth based on the reflectance difference between two bands. EVI is sensitive to areas with high biomass. GDVI, GEVI, and GNDVI focus on different bands and are sensitive to vegetation characteristics such as chlorophyll content. NDVI can reflect the greenness and biomass of vegetation. For visible images, the extracted red, green, and blue bands are normalized (Equations (1)–(3)) to mitigate reflectance variations between images. For multispectral images, the red, green, blue, and near-infrared (NIR) bands are processed. The formulas for calculating the vegetation index are shown in Table 2 and Table 3.

(1) $$r=\frac{r_{t}}{g_{t}+b_{t}+r_{t}}$$

(2) $$b=\frac{b_{t}}{g_{t}+b_{t}+r_{t}}$$

(3) $$g=\frac{g_{t}}{g_{t}+b_{t}+r_{t}}$$

#### 2.5.2. Extraction of Fruit Tree Structural Feature Ratio

The proportions of fruits, leaves, and flowers within a fruit tree significantly influence its fruit yield [40,41]. In this study, the structural feature ratios of fruit trees were extracted, including the flower area ratio during the flowering stage (f_1_), the flower-to-leaf area ratio during the flowering stage (f_2_), the fruit (covered with protective bags) area ratio during the young fruit stage (f_3_), and the fruit-to-leaf area ratio during the young fruit stage (f_4_). The extraction method was as follows. First, the entire fruit tree image was divided into 512 × 512 pixel patches. Then, these patches were processed using a pre-trained, high-accuracy semantic segmentation model to identify the flowers, fruits, leaves, and branches. Afterward, the pixel count for each category within each patch was recorded. Using these data, the proportions of different structural features of the fruit tree were calculated based on the total pixel count. The specific calculation formula can be found in Table 4.

#### 2.5.3. Feature Screening Methods

The Pearson correlation coefficient (PCC) is a statistical metric that measures the strength and direction of the linear relationship between two variables, ranging from −1 to 1. In this study, Origin software (version 2021) was used to perform statistical correlation analysis among fruit tree structural feature ratios, vegetation indices, relative chlorophyll content, and apple yield.

### 2.6. Selection of Apple Yield Estimation Model

In this study, four machine learning algorithms—k-nearest neighbors (KNN), partial least squares (PLS), random forest (RF), and support vector machine (SVM)—were employed to construct the apple yield estimation model.

KNN is an instance-based supervised learning algorithm for classification and regression. It makes predictions based on the outputs of the k-nearest neighbors in the feature space. For apple yield estimation, KNN predicts the yield by averaging the yields of the K most similar samples.

PLS is particularly effective for datasets with multicollinearity, making it ideal for high-dimensional, highly correlated data like the relationships between chlorophyll content, fruit tree structural features, and vegetation indices. In this study, PLS helped build an accurate yield prediction model by identifying latent components that explain the variance in both independent and dependent variables.

RF is an ensemble learning algorithm that improves prediction accuracy and model stability by constructing multiple decision trees. It is well-suited to high-dimensional data, exhibits strong resistance to noise, and effectively handles variability caused by environmental factors or measurement errors during field data collection. Additionally, RF can capture nonlinear feature interactions, making it well-suited to the complex patterns often found in agricultural data.

SVM is a powerful method for regression tasks, especially when dealing with nonlinear relationships in the data. SVM excels with small datasets, focusing on model generalization rather than overfitting the training data. By selecting appropriate kernel functions, SVM can adapt to nonlinear relationships, such as those between the proportion of flower areas at the flowering stage and the proportion of fruit areas at the young fruit stage. SVM’s ability to capture complex patterns in smaller datasets makes it an ideal choice for cases where data are limited or when the underlying relationships are highly nonlinear.

In this study, a total of 240 data samples were collected, including 80 samples of relative chlorophyll content (SPAD values), 80 samples of vegetation indices, and 80 samples of fruit tree structural feature ratios. These datasets were subsequently partitioned into a training set and a test set at a 7:3 ratio for model development and evaluation. By integrating these models and taking advantage of the advantages of each model to deal with the complexity of apple yield prediction, the SHAP interpretable artificial intelligence method was applied to reveal the importance of each feature to yield.

### 2.7. Model Evaluation Index

#### 2.7.1. Fruit Tree Structure Feature Segmentation Accuracy Evaluation Index

The accuracy of deep learning semantic segmentation was evaluated using three metrics: Mean Intersection over Union (MIoU), Pixel Accuracy (PA), and Class Pixel Accuracy. MIoU emphasizes the model’s performance at segmentation boundaries, PA measures the overall pixel-level classification accuracy, and Class Pixel Accuracy assesses the pixel-level classification accuracy for each individual class.

#### 2.7.2. Evaluation Index of Orchard Yield Estimation Model

The performance of the orchard yield estimation model was evaluated based on two metrics: the Root Mean Square Error (RMSE) and the coefficient of determination ($R^{2}$). A higher $R^{2}$ value (closer to 1) and a lower RMSE value (closer to 0) indicate better model performance and more accurate predictions. The calculation formulas for $R^{2}$ and RMSE are given as follows:

(10) $$R^{2}=1-\frac{{\sum_{l=1}^{m}\left(\overset{\wedge }{y_{l}}-y_{l}\right)}^{2}}{\sum_{l=1}^{m}{\left(\bar{y_{l}}-y_{l}\right)}^{2}}$$

(11) $$RMSE=\sqrt{\frac{1}{m}\sum_{l=1}^{m}(y_{l}-\overset{\wedge }{y_{l}}{)}^{2}}$$

In the formula, l represents the l_th sample, m represents the total number of samples, and $y_{l}$ represents the true value of each sample. ${\hat{y}}_{l}$ represents the predicted value of each sample. ${\bar{y}}_{l}$ represents the average true value of each sample.

## 3. Results

### 3.1. Fruit Tree Structural Feature Segmentation Results and Analysis

**Figure 4:**
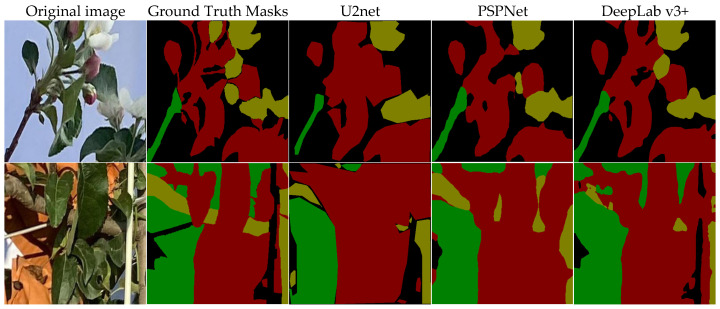
Results of fruit tree structural feature segmentation with different models. Flowering period image: G-branch, R-lea, Y-flower. Young fruit stage image: G-fruit, R-leaf, Y-branch.

The experimental results of fruit tree structural feature segmentation are presented in Table 5 and Figure 4. As shown in Table 5, the DeepLabv3+ model achieved a Mean Intersection over Union (MIoU) that was 0.11 and 0.03 higher than those of the U2Net and PSPNet models, respectively, demonstrating superior semantic segmentation performance. From Figure 4, it is evident that the U2Net model misclassifies fruit and trunk areas as leaves and exhibits limited ability to capture small-scale details, such as red flowers and twines. The PSPNet model misses the segmentation of red flowers and misclassifies white support rods as leaves, as well as misidentifying bagging as leaves. While the DeepLabv3+ model outperformed the other comparative models in overall segmentation accuracy, it still misidentified shadows as fruit categories when processing shadow regions of branches, indicating that the model’s feature recognition ability under varying lighting conditions requires further optimization. Consequently, DeepLabv3+ was selected as the base model for the fruit tree structural feature segmentation task.

**Figure 5:**
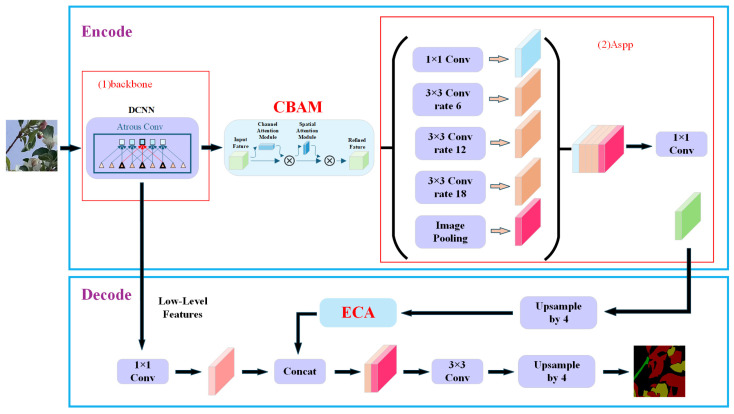
CBAM-ECA-DeepLabv3+ network model.

To address the limitations of DeepLabv3+ in processing shadow regions, this study proposes a dual attention enhancement architecture. First, the Convolutional Block Attention Module (CBAM) [42] was integrated at the end of the backbone network to enhance feature selection capabilities in both spatial and channel dimensions. Second, Efficient Channel Attention (ECA) [43] was embedded before the feature concatenation layer of the decoder to establish a cross-level feature association mechanism. The resulting CBAM-ECA-DeepLabv3+ network architecture is illustrated in Figure 5.

**Figure 6:**
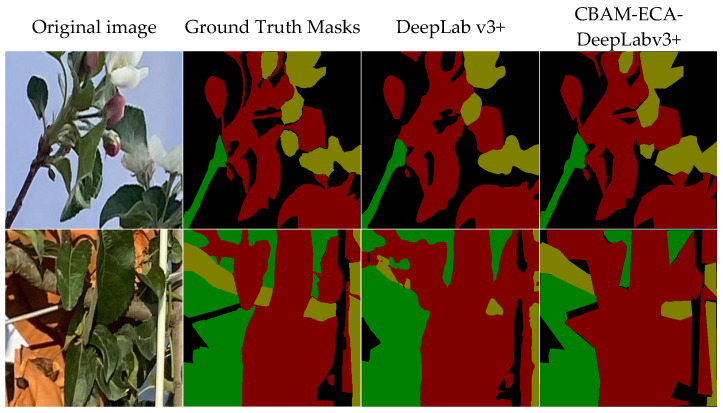
The segmentation effect of the improved model. Flowering period image: G-branch, R-lea, Y-flower. Young fruit stage image: G-fruit, R-leaf, Y-branch.

**Figure 7:**
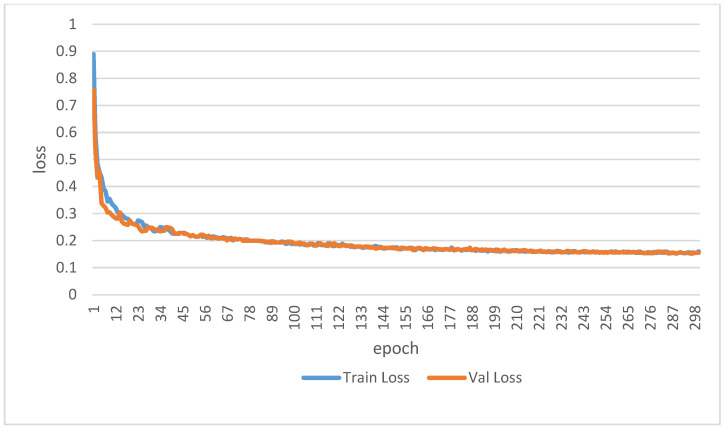
Training and validation loss curves of CBAM-ECA-DeepLabv3+ model.

The training results of the improved model are presented in Table 6. Integrating the CBAM alone resulted in an increase of 0.01, 0.04, and 0.04 in Pixel Accuracy (PA), Class Accuracy (AccClass), and Mean Intersection over Union (MIoU), respectively, compared to the baseline DeepLabv3+ model. Similarly, the addition of the ECA module alone increased these three metrics by 0.02, 0.04, and 0.05. Utilizing the dual-module joint optimization yielded the optimal performance (PA = 0.96, AccClass = 0.94, MIoU = 0.89), representing improvements of 0.04, 0.05, and 0.08 over the basic model, respectively. The visualization results are depicted in Figure 6. The improved CBAM-ECA-DeepLabv3+ model further enhanced the edge segmentation accuracy of small-scale targets, such as red flowers and young fruits, and effectively distinguished the semantic features of branches and shadow regions, thereby reducing the error segmentation rate. Figure 7 shows the training and validation loss curves of the CBAM-ECA-DeepLabv3+ model over 300 epochs. After approximately 200 epochs, both losses converge to around 0.1 with minimal difference between them, indicating stable training and no signs of overfitting. Table 7 presents the IoU values for each category. Leaves achieved the highest IoU (0.94), while branches showed the lowest (0.83). The IoU values for flowers and branches are slightly lower, which can be attributed to their smaller size and more complex boundaries. These results suggest that there is still room for further optimization in segmenting these finer structures.

### 3.2. Correlation Analysis and Multi-Source Feature Screening

The relative chlorophyll content of fruit trees at flowering stage ($s_{1}$) and young fruit stage ($s_{2}$), visible light and multispectral vegetation index, and the ratio of structural characteristics of fruit trees ($f_{1}$, $f_{2}$, $f_{3}$, $f_{4}$) extracted were correlated with apple yield by Pearson analysis, and the results are shown in Table 8.

According to Table 8, the Pearson correlation coefficients between the relative content of chlorophyll at the flowering stage ($s_{1}$) and the young fruit stage ($s_{2}$) and yield were 0.702 and 0.689, respectively, indicating a high correlation. In the multispectral vegetation index, the correlation coefficients of DVI ($d_{1}$), GDVI ($d_{2}$), and GEVI ($d_{3}$) with apple yield were higher than 0.6. Among visible vegetation index, only VARI ($k_{1}$) had the highest correlation coefficient with apple yield (0.755). The absolute values of the Pearson correlation coefficients between fruit structural feature ratios ($f_{1}$, $f_{2}$, $f_{3}$, $f_{4}$) and yield were all greater than 0.7, indicating a strong correlation. Therefore, the above characteristic parameters are selected as input variables for the yield estimation model.

### 3.3. Construction and Analysis of Orchard Apple Yield Estimation Model

#### 3.3.1. Construction of Production Estimate Model

The partitioned datasets were fused using various methods to create different feature sets (Table 9). These feature sets were then used to train the apple yield estimation model, and their impact on model accuracy was subsequently analyzed.

#### 3.3.2. Performance Analysis of Single Source Feature Modeling

The accuracy of the yield estimation models constructed using single feature categories is presented in Table 10. The R^2^ values of the four modeling algorithms ranged from 0.439 to 0.862. Using feature set A as the model input resulted in relatively low estimation accuracy, with the KNN model performing the least effectively (test set R^2^ = 0.439). After using feature set B, the performance of all models improved, particularly the support vector machine (SVM) model, which achieved a good estimation effect with this feature set (test set R^2^ = 0.860, RMSE = 19.688 kg), demonstrating the effectiveness of feature set B for yield estimation. Feature set C yielded the highest yield estimation accuracy, with the Random Forest (RF) model exhibiting the best performance (test set R^2^ = 0.862, RMSE = 18.735 kg), slightly surpassing the optimal result obtained with feature set B.

#### 3.3.3. Performance Analysis of Two-Source Feature Fusion Modeling

The accuracy of each model using two-class feature fusion for apple yield estimation is presented in Table 11. When feature set E is used as the model input, the R^2^ values of all models are improved, with the PLS model achieving an R^2^ of 0.916. Using feature set F as the model input resulted in lower performance across all models compared to feature sets D and E. The KNN model exhibited the lowest accuracy, with an R^2^ of 0.667.

#### 3.3.4. Performance Analysis of Multi-Source Feature Fusion Modeling

The accuracy of each model for yield estimation using multi-source feature fusion is presented in Table 12. The estimation accuracy of all four models with feature set G as the input parameters was superior to that of the other feature sets. The SVM model exhibited the highest estimation accuracy, with an R^2^ of 0.942 and an RMSE of 12.980 kg. Conversely, the KNN model demonstrated the lowest estimation accuracy, with an R^2^ of 0.764 and an RMSE of 23.957 kg.

**Figure 8:**
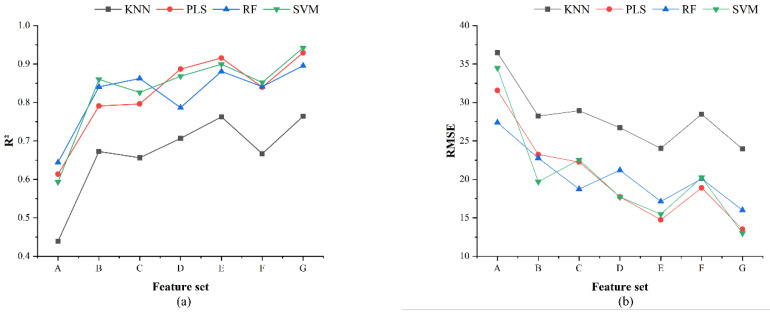
Precision comparison of apple yield estimation models under different feature datasets: (**a**) $R^{2}$; (**b**) RMSE.

The precision comparison of each model under different feature sets is illustrated in Figure 8. Compared to models using single and dual-feature sets, models incorporating relative chlorophyll content, vegetation indices, and fruit tree structural features demonstrated higher accuracy in orchard yield estimation. Generally, the R^2^ values of all models increased with an increasing number of features. Specifically, the SVM model’s orchard yield estimation accuracy with feature set G improved by 34.9%, 8.2%, 11.6%, 7.4%, 4.3%, and 9%, respectively, compared to the other six feature sets.

**Figure 9:**
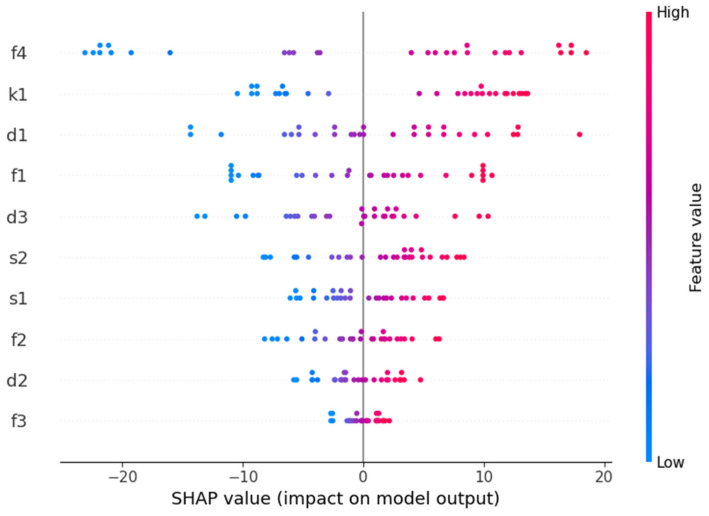
Importance of features that influence model output (based on SHAP values).

The SVM model is the optimal model for estimating the apple yield in small plots. To more accurately explore the importance of different features in predicting apple yield, the SHAP method was used to perform an interpretability analysis on the input features of the SVM model. As shown in Figure 9, the features F4, K1, and D1 have a significant impact on the model output, with feature F4 having the most pronounced effect. Higher feature values correspond to higher SHAP values, driving the model to predict higher yield estimates. In contrast, the SHAP value distributions for features F2 and F3 are more concentrated, indicating that these features have a smaller impact on the model’s predictions.

## 4. Discussion

This study proposes an apple yield estimation method based on multi-source feature fusion, including tree structure ratios, vegetation indices, and SPAD, aiming to improve the accuracy and robustness of apple yield prediction. To obtain various tree structure ratios, three base models—U2Net, PSPNet, and Deeplabv3+—were compared and analyzed. The Deeplabv3+ model, which demonstrated the best segmentation performance, was further improved by incorporating attention modules (CBAM and ECA) to enhance the accuracy of tree structure feature extraction. Experimental results show that U2Net and PSPNet models exhibit poor performance in capturing the structural details of apple trees under complex backgrounds. Deeplabv3+ improves segmentation accuracy by incorporating dilated convolution and ASPP modules [44], but the dilated convolution causes partial information loss, limiting its performance when dealing with complex structures and small targets. The improved CBAM-ECA-Deeplabv3+ model integrates CBAM and ECA modules, enhancing the model’s ability to focus on target regions and improve feature expression, thereby improving the capture of apple tree structure details. By cropping, the originally large target is converted into a smaller area, so that the model can be annotated and trained more finely. The segmentation accuracy of each category (flowers, fruits, leaves, branches) has a high IOU value, but there are also large differences between different categories, which are mainly affected by the target morphology, color contrast and detail complexity. The leaves have a high segmentation accuracy due to their large area and obvious texture characteristics; the fruits also achieve a high segmentation effect due to the obvious color difference caused by the bagging process. In contrast, the flowers are smaller and the details are complex, so the segmentation accuracy is lower, and the branches are also poor due to their lighter color, thin branches, and unclear transition from the background. Overall, the morphological regularity and color contrast of the target are the key factors affecting the segmentation accuracy.

When using the tree structure feature set B as input, the apple yield estimation model achieves higher accuracy. This suggests that tree structure feature ratios, derived from tree structure feature images extracted through image semantic segmentation algorithms, can serve as effective inputs for the yield estimation model. This may be because tree structure feature ratios are related to the allocation of photosynthetic products during different growth stages of the tree [45]. When the two types of features are fused, the yield estimation model based on feature set F (chlorophyll content + vegetation index) shows lower accuracy compared to those based on feature sets D (chlorophyll content + structural feature ratio) and E (structural feature ratio + vegetation index). This is likely due to the multicollinearity between relative chlorophyll content and vegetation index in feature set F, which reduces the model’s estimation capability [46]. The SVM model based on the fusion of three types of features achieves the best apple yield estimation accuracy. This is because the SVM model is well-suited to handling small datasets and excels in dealing with complex and nonlinear data, allowing it to effectively capture the intricate relationships between chlorophyll content, tree structure feature ratios, and vegetation index. The SVM model for small plot apple yield estimation shows that the $f_{4}$ feature (fruit-to-leaf ratio during the early fruit stage) has the greatest contribution, indicating a strong interaction between this feature and others, leading to greater variability in its impact on the model’s predictions. This finding is consistent with the study by Carella et al. [47]. The SHAP value distributions for features $s_{1}$ and $s_{2}$ (SPAD) are more concentrated, suggesting that these features have a more consistent influence on the model output, potentially indicating a more stable relationship.

In conclusion, by optimizing semantic segmentation models, the structural features of fruit trees can be accurately extracted. Combined with appropriate feature combinations and machine learning methods, efficient estimation of apple yield in orchards can be achieved, providing a new approach for yield estimation. In the training of the image semantic segmentation model, this study employed a dataset comprising images from the flowering and young fruit stages. These two stages exhibit significant differences in terms of color, texture, and structure, which increased the difficulty of model training and potentially affected convergence speed and segmentation accuracy. However, this approach also enhanced the model’s generalization ability. Future research could consider training images from the flowering and young fruit stages separately to optimize model performance. Although the yield estimation model has achieved satisfactory results in specific areas, it may not be fully applicable to other regions with different climates, varieties, and management practices. This could lead to overfitting or a decline in performance under diverse conditions, and its robustness still requires validation across a larger dataset. Additionally, the relatively small sample size used for SPAD value measurements and yield estimation, along with potential subjective factors in the selection process, could limit the generalizability of the findings. Future work will focus on expanding data sources, optimizing model parameters, exploring new algorithms, and further improving the generalizability and accuracy of fruit yield estimation models.

## 5. Conclusions

This study presents a multi-source feature fusion framework for apple yield estimation, integrating tree structural features, vegetation indices, and leaf chlorophyll content (SPAD). The CBAM-ECA-DeepLabv3+ model, incorporating attention mechanisms, achieved improved segmentation accuracy (mIoU = 0.89, 8% higher than the baseline), enabling the precise extraction of fruit tree structural features. The support vector machine (SVM) model, trained on a fused dataset integrating fruit tree structural feature ratios, vegetation indices, and SPAD values, achieved high yield estimation accuracy (R^2^ = 0.942; RMSE = 12.980 kg), with the fruit-to-leaf ratio identified as a key predictor. This approach enhances prediction robustness for small-scale orchards, providing a practical tool for precision agriculture. Future work will focus on expanding datasets and refining algorithms to improve generalizability across diverse orchard conditions.

## Figures and Tables

**Table 1:** UAV and camera parameters.

Parameter	DJI M300 (P1)	DJI M210 (MS600 Pro)
Flight Altitude	30 m	30 m
Flight Speed	2 m s^−1^	5 m s^−1^
Forward Overlap	85%	80%
Sideways Overlap	80%	85%
Pixel Resolution	8192 × 5460	1280 × 960
Imaging Range	Visible Light	450 nm, 555 nm, 660 nm 710 nm, 840 nm, 940 nm

**Table 2:** Calculation method of visible light vegetation index.

Color Index	Symbol	Computational Formula	Reference
Visible light atmospheric resistant vegetation Index	VARI	$\left(g-r\right)∕\left(g+r-b\right)$	[29]
Extra red vegetation Index	ExR	1.4r−g	[30]
Extra green vegetation Index	ExG	2g−r−b	
Green leaf vegetation Index	GLI	$\left(2g-b-r\right)∕\left(2g+b+r\right)$	[31]
Normalized Difference Index	NDI	$\left(g-r\right)∕\left(g+r\right)$	[32]
Modified green–red vegetation Index	MGRVI	$\left(g^{2}-r^{2}\right)∕\left(g^{2}+r^{2}\right)$	[33]

**Table 3:** Calculation method of multispectral vegetation index.

Color Index	Symbol	Computational Formula	Reference
Difference Vegetation Index	DVI	NIR−R	[34]
Green Difference Vegetation Index	GDVI	NIR−G	[35]
Enhanced Vegetation Index	EVI	$\left(2.5\times \left(NIR-R\right)\right)∕\left(NIR+6.0\times R-7.5\times B+1\right)$	[36]
Green Enhanced Vegetation Index	GEVI	$\left(2.5\times \left(NIR-G\right)\right)∕\left(NIR+6.0\times G-7.5\times B+1\right)$	[37]
Normalized Difference Vegetation Index	NDVI	(NIR−R)∕(NIR+R)	[38]
Green Normalized Difference Vegetation Index	GNDVI	(NIR−B)∕(NIR+B)	[39]

**Table 4:** Calculation formula of fruit tree structural characteristic ratio.

Fruit Tree Feature Information	Calculation Formula	Equation Number
Total canopy area in flowering stage images ($S_{A}$)	$S_{A}=F_{A}+I_{A}+G_{A}$	(4)
Total canopy area in young fruit stage images ($S_{B}$)	$S_{B}=F_{B}+I_{B}+G_{B}$	(5)
Flower area ratio during flowering stage ($f_{1}$)	$f_{1}=F_{A}/S_{A}$	(6)
Flower-to-leaf area ratio during flowering stage ($f_{2}$)	$f_{2}=F_{A}/I_{A}$	(7)
Fruit (bagging) area ratio during young fruit stage ($f_{3}$)	$f_{3}=F_{B}/S_{B}$	(8)
Fruit-to-leaf area ratio during young fruit stage ($f_{4}$)	$f_{4}=F_{B}/I_{B}$	(9)

$F_{A}$—flower area (flowering); $I_{A}$—leaf area (flowering); $G_{A}$—trunk area (flowering); $F_{B}$—fruit area (young fruit); $I_{B}$—leaf area (young fruit); $G_{B}$—trunk area (young fruit).

**Table 5:** Comparison of performance of fruit tree image segmentation models.

Split Object	Model Name	Evaluation Index		
		PA	AccClass	MIoU
Fruit tree image	U2net	0.86	0.79	0.70
	PSPnet	0.86	0.85	0.78
	DeepLabv3+	0.92	0.89	0.81

**Table 6:** Performance comparison of improved fruit tree image segmentation model.

Split Object	Model Name	Evaluation Index		
		PA	AccClass	MIoU
Fruit tree image	DeepLabv3+	0.92	0.89	0.81
	ECA-DeepLabv3+	0.93	0.93	0.85
	CBAM-DeepLabv3+	0.94	0.93	0.86
	CBAM-ECA-DeepLabv3+	0.96	0.94	0.89

**Table 7:** IoU results for different categories based on the CBAM-ECA-DeepLabv3+ model.

Class	Flowers	Fruits	Leaves	Branches	Mean
**IoU**	0.86	0.90	0.94	0.83	0.89

**Table 8:** Pearson correlation analysis.

Parameter	Correlation Coefficient	Parameter	Correlation Coefficient	Parameter	Correlation Coefficient
$s_{1}$	0.702	EXGR	0.024	EVI	0.429
$s_{2}$	0.689	GLI	0.437	RVI	0.505
MGRVI	0.445	DVI	0.687	$f_{1}$	0.792
RGBVI	0.186	GDVI	0.642	$f_{2}$	0.750
EXR	0.245	GEVI	0.602	$f_{3}$	0.740
NDI	0.353	NDVI	0.398	$f_{4}$	0.800
VARI	0.755	GRVI	0.501		
EXG	0.423	GNDVI	0.742		

**Table 9:** Feature set division.

Serial Number	Feature Set ID	Feature Parameters
1	A	$s_{1}$ $,s_{2}$
2	B	$f_{1}$ $,f_{2}$ $,f_{3}$ $,f_{4}$
3	C	$d_{1}$ $,d_{2}$ $,d_{3}$ $,k_{1}$
4	D	$s_{1}$ $,s_{2}$ $,f_{1}$ $,f_{2}$ $,f_{3}$ $,f_{4}$
5	E	$f_{1}$ $,f_{2}$ $,f_{3}$ $,f_{4}$ $,d_{1}$ $,d_{2}$ $,d_{3}$ $,k_{1}$
6	F	$s_{1}$ $,s_{2}$ $,d_{1}$ $,d_{2}$ $,d_{3}$ $,k_{1}$
7	G	$s_{1}$ $,s_{2}$ $,f_{1}$ $,f_{2}$ $,f_{3}$ $,f_{4}$ $,\;d_{1}$ $,d_{2}$ $,d_{3}$ $,k_{1}$

**Table 10:** Yield estimation accuracy of different algorithms with single source feature input.

Feature Set	Model	Test Set		Training Set	
		R^2^	RMSE	R^2^	RMSE
A	KNN	0.439	36.481	0.568	32.687
	PLS	0.614	31.574	0.676	27.741
	RF	0.644	27.406	0.638	30.638
	SVM	0.593	34.486	0.606	29.668
B	KNN	0.673	28.226	0.718	26.156
	PLS	0.791	23.230	0.883	16.681
	RF	0.841	22.761	0.861	16.589
	SVM	0.860	19.688	0.847	18.706
C	KNN	0.656	28.919	0.677	27.998
	PLS	0.796	22.260	0.781	23.051
	RF	0.862	18.735	0.916	14.161
	SVM	0.826	22.540	0.736	24.279

**Table 11:** Estimation accuracy of different algorithms with two-source feature fusion input.

Feature Set	Model	Test Set		Training Set	
		R^2^	RMSE	R^2^	RMSE
D	KNN	0.707	26.714	0.751	24.580
	PLS	0.887	17.723	0.872	17.180
	RF	0.787	21.207	0.866	18.595
	SVM	0.868	17.710	0.887	16.594
E	KNN	0.763	24.036	0.767	23.808
	PLS	0.916	14.749	0.904	15.071
	RF	0.881	17.121	0.916	14.290
	SVM	0.899	15.473	0.938	12.303
F	KNN	0.667	28.473	0.706	26.720
	PLS	0.840	18.899	0.854	18.862
	RF	0.841	20.075	0.874	17.189
	SVM	0.852	20.240	0.848	18.633

**Table 12:** Yield estimation accuracy of different algorithms with multi-source feature fusion input.

Feature Set	Model	Test Set		Training Set	
		R^2^	RMSE	R^2^	RMSE
G	KNN	0.764	23.957	0.775	23.382
	PLS	0.929	13.510	0.915	14.217
	RF	0.896	16.002	0.930	13.081
	SVM	0.942	12.980	0.936	11.989

## Data Availability

The original contributions presented in the study are included in the article, further inquiries can be directed to the corresponding author.
